# Clades, Grades, and the Genus Problem: A Case for Revising Hominin Taxonomy

**DOI:** 10.1002/ajpa.70344

**Published:** 2026-08-18

**Authors:** Ian Towle

**Affiliations:** ^1^ Biomedicine Discovery Institute, Department of Anatomy and Developmental Biology Monash University Melbourne Australia

**Keywords:** *Australopithecus*, genus Homo, Hominina, hominins, human taxonomy, *Paranthropus*

## Abstract

Taxonomic practice in evolutionary biology increasingly prioritizes the recognition of monophyletic groups (clades), yet paleoanthropology still applies this principle inconsistently. Here, I outline these inconsistencies and broader challenges in hominin taxonomy and propose a potential solution. *Australopithecus* is widely considered non‐monophyletic. In addition, the temporal depth of *Homo* is shallow relative to most primate genera, with recently diverged genera often taxonomically unstable due to complex morphological and genetic signatures and extensive gene flow among closely related taxa. The hominin fossil record exhibits pronounced morphological mosaicism and homoplasy, with supposedly key traits increasingly blurred both within and among genera. This obscures putative adaptive distinctions and contributes to persistent taxonomic disagreement. One potential solution is the subsumption of *Australopithecus* and *Paranthropus* within an expanded genus *Homo*. Such a revision would help address problems of non‐monophyly, avoid conceptual difficulties associated with genera with shallow time depths, and facilitate more coherent evolutionary comparisons within a more stable taxonomy. Within this framework, Hominini is best defined as the *Pan*‐*Homo* clade, with Hominina retained as the appropriate subtribal designation for the human clade. Although similar proposals have been advanced previously, accumulating fossil and genetic evidence makes this a timely moment to reconsider hominin taxonomy. This proposal has limitations, including the loss of taxonomic information relating to adaptive and ecological variation, particularly within *Paranthropus* and large‐brained *Homo*. At a minimum, however, this article argues for greater consistency and transparency in distinguishing clades from grades in homininan taxonomy, and for more consistent criteria for the recognition of genera.

## 1 Introduction

Clades, groups comprising a common ancestor and all of its descendants, are now the dominant framework for interpreting phylogeny and organizing biological taxonomy across most areas of the life sciences. Overall, biological anthropologists were not particularly fast or consistent early adopters of this approach, although some researchers embraced cladistic thinking relatively early. Indeed, from the 1970s onward, cladistic methods became increasingly influential within the field (e.g., Eldredge and Tattersall 1975; Delson et al. 1977; Bonde 1977; Skelton et al. 1986; Chamberlain and Wood 1987; Stringer 1987; Groves 1989). By the 1990s and 2000s, clade‐based analyses and discussions had become commonplace in paleoanthropology, although critiques persisted, particularly concerning the implementation and interpretation of cladistic methods (Brace 1981; Habgood 1989; Trinkaus 1990; Asfaw et al. 1999; Hawks 2004).

A central principle in Hennig's (1966) formulation of phylogenetic systematics is that taxonomy should reflect evolutionary relationships, such that groups at all taxonomic levels are monophyletic (i.e., clades). Although some researchers continue to express reservations about aspects of cladistic methodology or its practical application, most (but not all) paleoanthropologists would now agree that monophyly should be the goal for all taxonomic groupings. It is therefore striking that phylogenetic reconstructions routinely recover one or more of the commonly recognized Hominina genera (*Homo*, *Australopithecus*, and *Paranthropus*) as non‐monophyletic (here, the subtribe Hominina is used to refer to taxa more closely related to *Homo* than *Pan*; see Section 3).

In the past, ‘phenetic’ and ‘evolutionary’ taxonomies often recognized paraphyletic taxa, usually explicitly, in order to reflect evolutionary grades, or perceived differences in evolutionary tempo, rather than strict patterns of common descent (see discussion of this in Grine 1981; Groves 1989; Strait et al. 1997). This formed the basis of extensive debates between the 1960s and 1990s, when many researchers resisted replacing grade‐based classifications with clade‐based approaches. Influential figures such as George Gaylord Simpson and Ernst Mayr argued against cladistic methods, frequently using homininan evolution as a key example in support of retaining grade‐based taxonomy. Today, however, such debates have largely subsided. Clade‐based classification is not only the dominant taxonomic framework across the biological sciences, but is also effectively required in contemporary systematic practice in most fields.

Contemporary phylogenetic studies generally seek to reconstruct evolutionary relationships in a manner consistent with the goals of cladistics, identifying clades and inferring patterns of common ancestry. In recent years, model‐based approaches have become increasingly prominent, complementing and in some cases surpassing parsimony analyses in frequency of use. These methods employ explicit evolutionary models to estimate the most probable phylogenetic trees, with Bayesian inference emerging as the most widely used approach in homininan phylogenetics (see Gousset et al. 2026). They have been applied to a range of data types, including geometric morphometric data and discrete morphological characters, the latter often resembling those used in traditional cladistic analyses (Organ et al. 2011; Dembo et al. 2015; Parins‐Fukuchi et al. 2019; Irish and Grabowski 2021). It has also become increasingly common to combine parsimony‐based and model‐based methods within the same study (Argue et al. 2017; Mongle et al. 2019, 2023; Martin et al. 2021). Lastly, principal components analysis (PCA) has also become widely used to support systematic interpretations in homininan studies, with proximity in morphospace often taken as evidence of phylogenetic affinity (e.g., Harvati et al. 2019; Hershkovitz et al. 2021; Davies et al. 2024; but see Raskin et al. 2026).

At the same time, cladistic analyses continue to be widely employed either independently or alongside other phylogenetic approaches (e.g., Martinón‐Torres et al. 2007; Argue et al. 2009; Berger et al. 2010; Irish et al. 2013; Zeitoun et al. 2016; DeSilva et al. 2019; Simon‐Maciejewski et al. 2024). Consequently, although debate persists regarding the most appropriate methods to use, the past two decades have seen the emergence of a near‐universal consensus that phylogenetic analyses should seek to reconstruct evolutionary relationships and that taxonomy should reflect those relationships. As discussed below, however, the implementation of this principle in homininan taxonomy has not kept pace with its widespread theoretical acceptance.

In particular, the paraphyly of *Australopithecus* has long been recognized (Harrison 1993; Strait et al. 1997; Wood and Richmond 2000; Grine et al. 2006; Strait 2010; Kimbel 2014; Sekhavati et al. 2025). This issue may not be apparent to newcomers to the field; however, because most studies, including those that explicitly employ or discuss clade‐based approaches, continue to use the genus uncritically. Although the specific relationships vary among phylogenetic analyses, the overall pattern is consistent: one or more *Australopithecus* taxa are recovered as more closely related to *Homo* and/or *Paranthropus* than to other *Australopithecus* taxa (see below). A straightforward response to this paraphyly would be to split *Australopithecus* into multiple genera. However, this approach tends to generate numerous monotypic genera, likely requiring more splitting than simply recognizing genera such as *Praeanthropus* and *Kenyanthropus*, and thereby reducing the practical utility of the classification. At the same time, the rapid expansion of the homininan fossil record over recent decades has produced numerous unexpected discoveries that have undermined many of the traits once considered diagnostic, or at least important, for particular genera and their presumed adaptive niches. Consequently, *Australopithecus* continues to function largely as a non‐monophyletic “wastebasket” grade rather than a well‐defined evolutionary clade. Similar issues have also been raised regarding the genus *Homo*, whose diagnosis and taxonomic boundaries remain the subject of substantial debate (see Zanolli et al. 2026).

In this article, I review several longstanding issues in homininan systematics and examine how accumulating evidence from diverse sources, including new fossil discoveries, genetic data, phylogenetic analyses, and comparative studies of living primates, has amplified problems that have long existed within homininan taxonomy. In recent years, these issues have become increasingly difficult to ignore as new discoveries continue to challenge traditional classifications. I propose a taxonomic framework that brings the human clade into closer alignment with clade‐based principles, drawing on both the expanded homininan fossil record and comparisons with other primate groups.

## 2 Expanding the Genus *Homo*

In recent decades, the emphasis on ensuring that genera are monophyletic has rendered many earlier genus definitions, based more purely on grades, perceived differences in evolutionary rates, diet, ecological niche, or behavior, invalid or in need of revision. Nevertheless, because assigning taxa to higher taxonomic ranks retains an inherently arbitrary element, judgment calls are still required to determine when a genus is an appropriate category (i.e., how many species to include). In this process, several factors are typically considered, often in combination, including time since common ancestry, genetic distances, ecological niche or adaptive zone distinctiveness, and reproductive isolation (i.e., the lack of hybridization). Therefore, for a workable definition of the genus *Homo* (and other homininan genera), it must first and foremost be monophyletic and then ideally also justifiable on the basis of one or more of these additional criteria (see Wood and Collard 1999). Additionally, monotypic genera should also be avoided, when possible, as this adds no additional information (see Brower 2026). Therefore, an additional criterion would be to limit the number of monotypic genera. Based on these considerations, I first explore potential insights from the fossil record, followed by evidence from living primates.

### 2.1 Fossil Evidence

Today, there is mostly consensus regarding the composition of the genus *Homo*, at least in the sense it is restricted to a single extant species ( *Homo sapiens* ), spans a relatively short interval in the fossil record (generally estimated at ~2–3 Ma), and includes a largely agreed upon set of fossil taxa (with some ongoing debate for particular early taxa, see below). However, in the past there have been much broader proposals for *Homo*, ranging from expansive suggestions that would include all African great apes, or particular ones, within *Homo*, to definitions encompassing all Hominina (e.g., Mayr 1950; Goodman 1999; Watson et al. 2001; Uddin et al. 2004).

The first fossil remains attributed to *Homo* did not appear until the second half of the nineteenth century, with the inclusion of Neanderthals. The genus subsequently expanded with the discovery of fossils now assigned to *Homo erectus* and *Homo heidelbergensis*, although many additional genera and species were initially proposed for particular sites and specimens before later consolidation into these taxa, most notably in the case of *H. erectus* (see Collard and Wood 2015). By the mid‐twentieth century, this process of consolidation had accelerated, leading eventually to the emergence of the three dominant homininan genera in use today (*Homo*, *Australopithecus*, and *Paranthropus*) and the subsuming of many earlier named genera into them (see Robinson 1962). While some researchers continued to employ legacy generic names and occasional new genera have been proposed since, these alternatives (or additions) have generally failed to gain widespread acceptance, except for potential earlier homininan genera discussed later. However, it should be noted that this was not a linear process, and the use of the genus *Paranthropus* in particular has waxed and waned over the last 50 years (see Grine 1981; Grine and Wood 2026).

The description of *Homo habilis* (Leakey et al. 1964) expanded the range of morphology and traits encompassed by the genus *Homo*, particularly with respect to acceptable brain size (Le Gros Clark 1955). Indeed, many researchers opposed its inclusion largely on this criterion alone, as cranial capacity had often served as a primary defining feature of the genus. Since then, several geographically widespread fossil samples with brain sizes overlapping, or only minimally larger than, those of *Australopithecus* (e.g., *Homo floresiensis*, some of the Dmanisi specimens, and *Homo naledi*) have been assigned to *Homo* (Brown et al. 2004; Berger et al. 2015; Rightmire et al. 2019). In parallel, the earliest proposed appearance of *Homo* has been pushed further back in the fossil record (see Villmoare et al. 2026). In this context, the defining features of the genus *Homo*, in terms of adaptive niche or shared behaviors, but also key traits such as brain size, have slowly been eroded.

Some analyses have recovered *H. floresiensis* and *H. naledi* as more closely related to certain *Australopithecus* taxa than to other members of *Homo* (Caparros and Prat 2021; Gousset et al. 2026). Other studies have placed these species in close association with early *Homo* taxa, including *H. habilis* , or recovered them in basal positions within *Homo* (e.g., Dembo et al. 2015; Argue et al. 2017; Irish and Grabowski 2021). In contrast, alternative analyses have identified closer affinities with later members of the genus, particularly *H. erectus* (Zeitoun et al. 2016; Dembo et al. 2016). As a result, the phylogenetic placement of both taxa remains uncertain. This uncertainty has important taxonomic implications. Proposals to remove early *Homo* taxa such as *H. habilis* from *Homo* would now create additional challenges, especially regarding the classification of taxa such as *H. floresiensis* and *H. naledi*, whose positions within (or outside) the *Homo* clade remain unresolved. Any substantial revision of genus level taxonomy must therefore account not only for early *Homo* taxa but also the growing number of later specimens/taxa, which continue to occupy unstable positions across competing phylogenetic analysis. There is also the ongoing, and perhaps increasing, possibility that early *Homo* taxa as currently defined derive from different *Australopithecus* taxa, which would render *Homo* non‐monophyletic even before these much later small‐brained taxa are considered (see Zanolli et al. 2026).

Many, likely most, paleoanthropologists currently regard the species commonly assigned to *Paranthropus* ( *P. robustus* , *P. boisei*, and *P. aethiopicus* ) as forming a monophyletic group, thereby justifying their recognition as a distinct genus characterized by robust craniodental morphology and a specialized diet focused on tough and/or hard foods (Wood and Schroer 2017; Towle et al. 2025; Strait et al. 2026). However, this conclusion is likely far from settled. Ongoing debate centers on whether the southern and eastern African forms evolved independently from different *Australopithecus* lineages. If so, *Paranthropus* would not constitute a clade but instead represent regionally derived megadont taxa (see Wood and Lonergan 2008; Wood and Schroer 2017; Strait 2013). Moreover, even if the monophyly of *Paranthropus* were confirmed, its placement within the broader homininan radiation would still raise classificatory challenges for both *Homo* and *Australopithecus*, depending on how this lineage is resolved within the wider homininan phylogeny.

The situation is further complicated by the existence of a gradient of dental “robustness” among homininans. Some *Australopithecus* species, including *A. africanus* , have long been recognized as approaching the dental robustness observed in some *Paranthropus* taxa/specimens (White et al. 1983). In recent decades, this picture has become even more complex, with *Australopithecus garhi* exhibiting larger teeth than even the most megadont *Paranthropus*, *P. boisei*, yet it is still assigned to *Australopithecus* based on other characteristics (Asfaw et al. 1999; but see Wood and Schroer 2017). Certain supposed early *Homo* specimens also show affinities with *Paranthropus*, and for some fossils, debate has intensified over the appropriate generic assignment (Davies et al. 2020; Zanolli et al. 2022). Moreover, it has even recently been suggested that *Homo naledi* may be a late surviving, dentally reduced, *Paranthropus* lineage (Zanolli 2026).

The traditional view of *Paranthropus* as a distinct evolutionary lineage specialized for consuming hard foods has gradually been eroded from multiple directions. In particular, growing evidence suggests that the eastern and southern African *Paranthropus* taxa had substantially different diets, neither of which appears to have relied heavily on hard food items (Cerling et al. 2011; Towle et al. 2021; Constantino et al. 2026; but see Strait 2026). This does not undermine the possibility that *Paranthropus* represents a clade. Shared dental and cranial traits may still reflect common ancestry, even if these features were subsequently employed in slightly different ecological contexts. Nevertheless, these findings increasingly weaken the functional justification traditionally used to distinguish *Paranthropus* as a separate genus. All this said, somewhat paradoxically, *Paranthropus* may be both the most controversial (most discussed anyway) and the strongest of the currently recognized homininan genera. Indeed, it remains one of the few (or even only) homininan genera for which there is at least a plausible case that can be made for both a clade and shared morphology corresponding to a distinctive behavioral niche (even if the actual foods consumed were somewhat different).

When *Australopithecus* is defined in a way that excludes one or more of its descendant lineages, the genus becomes paraphyletic. All recently published phylogenies containing multiple *Australopithecus* taxa recover a paraphyletic configuration (e.g., Irish et al. 2013; Dembo et al. 2015; Rocatti and Perez 2019; Püschel et al. 2021). As a result, the prevailing homininan taxonomic framework remains essentially grade‐based. This situation has become somewhat contradictory in recent decades, since despite the growing application of finding and describing clades in phylogenetic analyses, the taxonomic implications of the resulting paraphyletic trees are rarely addressed explicitly. In most other areas of biology, the commonly used three‐genus framework of *Australopithecus*, *Paranthropus*, and *Homo* would be abandoned, with taxa either subsumed into an expanded *Homo* or reclassified into additional (or different) genera to better reflect evolutionary relationships.

Although it is now rarely stated that *Australopithecus* is a “wastebasket” taxon, this view was explicitly promoted in the past, particularly before the widespread adoption of clade‐based taxonomies. For example, when specimens now typically assigned to *A. afarensis* were first discovered and described, a wide range of interpretations were proposed, including that they were (or resembled) *Ramapithecus*, *A. africanus* , early *Homo*, or *Homo erectus* (e.g., Leakey 1976; Boaz 1979; Olson 1981; Oxnard 1979). Ultimately, the proposal that all Hadar and Laetoli specimens (at that time) could be accommodated within a single species gained traction, a species now recognized as *Australopithecus afarensis*, and since extended to include fossils from other sites (Johanson and White 1979). These specimens were ascribed to the genus *Australopithecus* for the following reason: “Since these creatures did not exhibit brain expansion normally associated with the genus *Homo*, yet were clearly bipedal, they were assigned to *Australopithecus*” (White et al. 1983, 729). This use of *Australopithecus* defined by an adaptive grade of homininan has continued ever since (see discussion in Strait 2010; Kimbel 2014).

The problem is not solely cladistical. Behavioral and ecological distinctions once thought to separate these three genera have also eroded substantially in recent years, such that even grade‐based genera no longer appear defensible. *Paranthropus*, traditionally characterized as a hard‐object specialist lacking tool use, is now recognized as far more variable in diet, behavior, and temporal and ecological niche (Plummer et al. 2023; Alemseged et al. 2026). Individual species display divergent dietary adaptations, some of which may align more closely with other homininans than with one another (Ungar et al. 2008; Cerling et al. 2011; Wood and Patterson 2020; Towle et al. 2021). Traits long considered diagnostic of *Homo*, include increased brain size, often associated with stone tool technology, and the consumption of higher quality foods through processing (Antón et al. 2014). However, as additional specimens and taxa have been discovered in recent decades, it has become increasingly difficult to identify key features (that could justify genus level differences) unique to *Homo* that are not also present in *Paranthropus* or *Australopithecus* (Hawks 2018). Consequently, the prevailing generic framework fails on two fronts, it neither recovers monophyletic groupings nor identifies consistent ecological or behavioral distinctions that could theoretically justify maintaining separate genera.

Recent debates have focused primarily on whether material commonly assigned to *Homo habilis*, *Homo rudolfensis*, and early *Homo* more broadly from eastern and southern Africa should remain within *Homo*, be moved to *Australopithecus*, or be placed in a separate genus (Wood and Collard 1999; Collard and Wood 2015). These discussions further highlight the limitations of the current genus‐level framework. Difficulties in assigning specimens to *Homo* or *Australopithecus* have long been recognized, particularly regarding *H. habilis* , which some researchers argue may encompass multiple species, some of which are better placed within *Australopithecus* (Stringer 1986; Lieberman et al. 1988; Trinkaus 1990). However, attempts to shorten the temporal range of *Homo* may exacerbate rather than help resolve the problem. To evaluate this possibility and its implications for higher‐level taxonomic classification, it is instructive to examine analogous evidence from other catarrhine primate groups.

### 2.2 Living Primate Comparisons

The issues outlined above are well known within the paleoanthropological community. If the field is to align more closely with broader evolutionary biology, now is an opportune moment to develop more consistent taxonomic frameworks for our section of the great ape family tree. One productive approach is to draw comparisons with living non‐human primates. Many of the challenges discussed above, such as complex evolutionary histories and gene flow, are also present in other primate groups and have been assessed recently using comprehensive genetic analysis. Consequently, these taxa provide an ideal comparative context for evaluating how higher‐level taxonomic categories can be applied and interpreted more effectively to Hominina.

An approach proposed to standardize higher level taxonomy in primates, including at the genus level, is the application of fixed temporal thresholds to each taxonomic rank. The goal of such systems is to facilitate comparisons across groups and to avoid problems such as extensive intergeneric hybridization when genera are defined too recently, or excessive behavioral and ecological diversity when divergence times are placed too far back in the past. Several frameworks have been put forward to implement such temporal criteria in primate generic classification (e.g., Goodman et al. 1998; Groves 2001; Garbino 2015). The divergence intervals proposed have been quite broad, ranging from around 4 to 11 Ma (Goodman et al. 1998; Groves 2001; Wildman et al. 2003; Cameron and Groves 2004). Within each proposed system, a specific divergence point is then selected to delimit genera. Ideally, this node would correspond either to a well‐supported phylogenetic split or to the emergence of a major adaptive zone (e.g., ecological or behavioral specialization).

Although these systems offer advantages, they also entail important drawbacks. Most notably, a degree of arbitrariness remains in how genera are delimited, and many taxa do not conform to the expectation of a well‐defined divergence point within a given range. These limitations are particularly evident when applying time‐based approaches to genus level classification in great apes, where low species diversity among extant taxa and uncertainties in the fossil record complicate divergence estimates. For example, if a temporal window of 6–9 Ma is used to define genera, several complications arise. Depending on the divergence estimates adopted, this interval may or may not encompass the split between *Homo* and *Pan*, or the deeper divergence between *Gorilla* and the *Homo*‐*Pan* clade. Furthermore, incorporating humans together with one or both of the other African great ape clades into a single genus is problematic, as there is no compelling ecological or behavioral justification for collapsing one or both into *Homo*. Maintaining separate genera for each living great ape group remains the norm today, and still aligns with some of the lower temporal ranges (i.e., those ~4–7 Ma).

Time based approaches to defining genera have attracted relatively little support. Such structures present several additional difficulties. Most importantly, they can impose taxonomic groupings that convey little information about the morphology, ecology, or behavior that genera were traditionally intended to capture. They also require estimates of divergence times and common ancestry that are often highly variable. Furthermore, variation in generation times among lineages complicates attempts (or justification) to apply temporal thresholds consistently across taxa. Nevertheless, many researchers would agree that divergence time should not be ignored entirely when considering taxonomic classifications. Firstly, because a taxonomy has to be useful at multiple levels, having sister genera with widely different chronologies makes higher level categories challenging (see Section 3). This is also especially relevant in light of growing evidence for evolutionary processes such as hybridization and introgression, which can persist long after lineages have initially diverged. As a result, placing genus‐level boundaries too recently in time risks obscuring complex evolutionary relationships and creating unstable taxonomies if relationships within a particular radiation remain unresolved.

Until relatively recently, hybridization was usually interpreted as a failure of reproductive barriers, and suggesting that two taxa should be considered the same species (e.g., the Biological Species Concept), or in the context of producing unviable offspring. In recent decades, how common hybridization actually is has become increasingly clear and there is growing recognition of its role in shaping taxonomic diversity (Arnold 1992; Grant and Grant 2007; Abbott et al. 2013; Cortés‐Ortiz et al. 2019; Runemark et al. 2025). Naturally occurring hybridization has now been documented in all well studied primate groups (see Wall et al. 2016 and references therein). Primate genera most often span divergence times of approximately 5–10 Ma, but there are some with more recent common ancestry. These cases offer valuable parallels for understanding several of the taxonomic challenges encountered in the Hominina fossil record.

A well‐studied example is provided by baboons (*Papio*) and their close relatives (*Rungwecebus, Theropithecus*, and *Lophocebus*). This group is especially informative for comparison with homininans, as the estimated timing of their common ancestor broadly overlaps with the period during which *Australopithecus*, *Paranthropus*, and *Homo* diversified, and because these primates expanded into a wide range of, presumably, similar ecological contexts on the same continent. The large bodied African Papionini genera (*Mandrillus, Papio*, *Theropithecus*) were originally considered a monophyletic group, with mangabeys usually treated as a separate lineage. However, from the mid‐1970s onward, morphological and behavioral evidence started to suggest that mangabeys may actually be deeply embedded within the large bodied radiation (or the other way around depending on your perspective). Although typically placed in a single genus (*Cercocebus*), differences in ecology and behavior, as well as locomotion, led to the recognition of two distinct mangabey genera, a more terrestrial group (*Cercocebus*) and a more arboreal group (*Lophocebus*) (Groves 1978; Nakatsukasa 1996; McGraw 1998). Molecular studies have since confirmed that these groups are polyphyletic, with *Cercocebus* sister to *Mandrillus* and *Lophocebus* more closely related to *Papio* and *Theropithecus* (Disotell 1994; Harris and Disotell 1998; Fleagle and McGraw 1999).

The complexity of Papionini relationships increased further with the discovery of a new mangabey taxon (Jones et al. 2005). Molecular analyses soon demonstrated that its placement in *Lophocebus* made the genus diphyletic, since the new species was actually more closely related to baboons, leading to the requirement of a new genus, *Rungwecebus* (Davenport et al. 2006). It is possible that *Rungwecebus* may be nested within *Papio*, or originated through hybridization between *Papio* and *Lophocebus*, but currently the most likely scenario is that *Rungwecebus* is a distinct sister lineage to *Papio* that later experienced repeated gene flow with specific baboon taxa, producing complex genetic signatures (Burrell et al. 2009; Zinner et al. 2009, 2018; Roberts et al. 2010). Additional evidence for intergeneric hybridization within this clade has been documented, including between geladas and baboons (Jolly et al. 1997), and between the white‐naped mangabey ( *Cercocebus lunulatus* ) and the olive baboon ( *Papio anubis* ; Goodwin et al. 2022). Together, these cases indicate that extensive hybridization has likely occurred throughout much of the 4–5 Ma evolutionary history of this large clade. In this respect, the group presents challenges closely analogous to those faced in homininan taxonomy. Complex morphological signatures and rampant gene flow make relationships difficult to untangle in such rapid radiations. Similar complications and surprises should be expected in the similarly large homininan diversification.

At the species level, extensive hybridization has also been documented within these groups, particularly in *Papio*, with pervasive gene flow across taxa, including extensive admixture with extinct “ghost lineages” (Jolly et al. 2011; Zinner et al. 2013; Wall et al. 2016; Walker et al. 2017; Rogers et al. 2019). These patterns relate to the complex evolutionary history of African Papionini, which have experienced recurrent range shifts involving population fragmentation and subsequent secondary contact within a closely related, and large, radiation (Zinner et al. 2011; Fischer et al. 2019; Rogers et al. 2019). Although detailed genetic data are currently available only for relatively recent *Homo* populations, similar processes are clearly evident in homininans and presumably operated throughout earlier phases of homininan evolution in Africa. These closely related Papionini genera persist as separate genera today, rather than being subsumed into a single genus (*Papio*), likely because genetic data from living populations allow their evolutionary relationships to be assessed more directly. Even so, it could be argued that collapsing them into a broader genus might simplify discussions of complex cases such as *Rungwecebus* and intergeneric hybridization, both recent and historical. In homininans, this challenge is amplified by the lack of genetic data for most taxa/samples.

There are other examples of temporally recent genera relevant to this discussion. This includes other members of Cercopithecidae, such as the colobine genera *Nasalis* and *Simias*, which share a very recent common ancestor, likely within the last 2.5 million years (Roos et al. 2011; Liedigk et al. 2012). Studies on these groups indicate that substantial gene flow continued between the genera after their initial divergence, as evidenced by discrepancies between mitochondrial and nuclear DNA dating (Roos et al. 2011). Historically, researchers have suggested that *Simias* could, or perhaps should, be subsumed into the genus *Nasalis* (Delson 1975; Groves 1970; Brandon‐Jones et al. 2004; Whittaker et al. 2006; Roos et al. 2022), with the older studies typically based on morphology and more recent work utilizing genetic data. The main counterargument has focused on habitat differences, with some researchers suggesting separate genera is justified because of differences in ecological niche (Napier 1985; Oates et al. 1994). However, these assessments predate a more comprehensive understanding of proboscis monkey distribution, which is now known to be much broader across Borneo than previously thought (Meijaard and Nijman 2000; Matsuda et al. 2009; Bernard et al. 2025). Consequently, these genera may be more similar in adaptive strategy than previously recognized, including overlapping seasonal resource use (Bennett and Davies 1994; Whittaker et al. 2006). Regardless of whether these taxa are treated as a single genus or as separate, the key point is that temporally recent genus‐level boundaries are inherently prone to taxonomic instability. Furthermore, if a detailed fossil record existed for the last 2–3 million years (as is the case for homininans), distinguishing between these taxa would be challenging given gene flow was present for most of this period.

Hybridization is widespread among other colobines, including langurs (Wang et al. 2015; Al‐Razi et al. 2023; Singh et al. 2025) and has likely played a critical role in the evolutionary history of some colobines (see Roos et al. 2011). Hybridization has also influenced vervet evolution (Svardal et al. 2017), and guenons exhibit extensive hybridization both between closely related species and among more distantly related taxa, including forms now classified in different genera, documented through both genetic and behavioral studies (e.g., Aldrich‐Blake 1968; Detwiler et al. 2005; de Jong and Butynski 2010). Therefore, across Cercopithecidae, whenever detailed studies have been undertaken, complex gene flow is consistently observed among closely related taxa, including across genera, but typically only those that share a common ancestor within the last couple/few million years.

Moving closer to our part of the catarrhine family tree, other apes also provide valuable insights. Recent research has found evidence of extensive and complex hybridization and gene flow across all apes. For example, in *Pan*, gene flow persisted long after the initial bonobo‐common chimpanzee split, likely continuing over a sustained period within the last half million years (De Manuel et al. 2016). Of particular relevance for comparisons with homininan genera, the deepest split among extant orangutans (*Pongo*) occurred nearly 3.5 million years ago, followed by substantial historical and more recent gene flow (Nater et al. 2017). Gorillas offer another useful perspective, with a recent study identifying strong evidence for a now extinct ‘ghost’ lineage that diverged from the common ancestor of living gorillas around 3.5 million years ago and later interbred with the lineage leading to eastern gorillas, but not western gorillas (Pawar et al. 2023). Such ghost lineages have also been identified in later homininans, and it is possible (or perhaps likely) that substantial introgression from groups currently classified in other homininan genera could have contributed significant genetic material to particular *Homo* taxa. Taken together, this catarrhine comparative perspective suggests that any genus with a very short temporal range will be prone to be affected by complex patterns of introgression and hybridization.

Hybridization can also have diverse effects on speciation and lineage evolution. Gene flow and recombination can sometimes accelerate diversification through processes such as adaptive introgression (Abbott et al. 2013). Potential benefits, or at least the emergence of novel traits with adaptive potential, include access to new alleles, increased genetic diversity, and distinctive phenotypes, as observed in living Papionini (Jolly et al. 1997; Ackermann et al. 2014; Buck et al. 2025). Consequently, these processes may also have played an active role in homininan evolution, highlighting the importance of discussing taxonomy within a framework that accommodates such interactions. The most obvious way to achieve this is to give genera sufficient temporal depth so that stable taxonomies can be created and used.

### 2.3 A Potential Solution

Based on the issues and factors described above, both in the homininan fossil record and in studies of evolutionary complexities in other catarrhine primates, I suggest to broaden the definition and temporal scope of the genus *Homo* to include specimens currently attributed to *Paranthropus*, *Australopithecus*, as well as closely related taxa that have been assigned separate genera, but fall within the same radiation (most commonly used today being *Kenyanthropus*). This expansion remains consistent with standardized higher taxonomy time period frameworks, particularly at the lower ranges (i.e., 4–6 million years). Such a setup may represent a “Goldilocks” zone for defining our genus, as it would help resolve issues of non‐monophyletic groupings by forming what is currently typically (i.e., is relatively stable compared to most other potential nodes) represented as a clade, reduces complications arising from complex gene flow among closely related taxa, but avoids the challenge of incorporating *Pan* (and potentially *Gorilla*) into our genus if expanded further.

This approach would place the genus *Homo* in the 4–5 million‐year range. Examples from other primates suggest that attempts to recognize multiple genera within the last few million years of homininan evolution are likely to encounter numerous complications. While extending the temporal range does not eliminate these issues, it provides a framework in which the taxonomy can be interpreted more coherently, without necessitating continual redefinition of genera with each new fossil discovery, phylogenetic analysis, or behavioral/ecological inference.

Given continued debate over the placement of proposed earlier homininans (or even their placement within this subtribe), restricting the genus to currently recognized *Australopithecus*, *Homo*, and *Paranthropus* (and *Kenyanthropus*) represents a convenient and defensible (at present) clade. These earlier taxa (including *Sahelanthropus*, *Orrorin*, and *Ardipithecus*) are sometimes interpreted as fitting elsewhere within the ape family tree, and some key homininan traits (or more specifically later homininan traits) have been argued to be absent in these groups (e.g., Strait 2013; Macchiarelli et al. 2020; Wood et al. 2020; Williams et al. 2026). Therefore, although some, or all three, of these taxa may well be homininans, there is little evidence to suggest they are in the expanded *Homo* clade proposed here. Indeed, the proposed expanded *Homo* framework does not preclude these genera from being considered homininans, as *Homo* need not encompass the entirety of homininan evolution, and multiple genera may be justified within this broader clade. However, the proposed expanded *Homo* constitutes a well‐supported clade, which adding these additional taxa would undermine.

This contrasts with the current use of the genus *Homo*, for which considerable debate remains regarding both the identity of the earliest members of the genus and the taxa that may have given rise to it. Proposed direct, or near‐direct, ancestral candidates encompass a broad range of *Australopithecus* (and *Kenyanthropus*) taxa (Asfaw et al. 1999; Berger et al. 2010; Berger 2012; Kimbel and Villmoare 2016; Martin et al. 2024). Indeed, typically it is stated that this ancestor is a form of gracile australopith (Ledogar et al. 2025), yet at the same time many studies also find *Paranthropus* more closely related to *Homo*, further complicating this issue. Although ultimately there is general consensus that *Homo* did in some sense originate from the broad radiation that is currently represented by *Australopithecus*. Consequently, these specific interpretations remain controversial due to unresolved questions concerning both their chronological placement and the position of other homininans within this evolutionary framework (Kimbel 2014). By contrast, the base of a more inclusive *Homo* clade is considerably better resolved. In many recent phylogenetic analyses, *Australopithecus anamensis* is recovered as the sister taxon to a clade comprising all other *Australopithecus*, *Paranthropus*, and *Homo*. Consequently, this represents a plausible and broadly supported clade that is well suited for taxonomic recognition. Such a definition provides a relatively stable phylogenetic reference point for an expanded concept of *Homo* and is likely to generate less taxonomic uncertainty and debate than the current framework.

Although this proposal differs from those of Collard and Wood (2015), Tattersall (2017), and Villmoare (2018), it addresses the same underlying issues, albeit with different solutions. Tattersall (2017), for example, questions what type of genus is being justified when humans are grouped with fossil specimens such as the Ledi‐Geraru mandible (NME LD350‐1), whereas Villmoare (2018) argues that such inclusion is defensible under strict cladistics. Collard and Wood (2015) instead advocate re‐evaluating the definition of *Homo* and identifying more robust diagnostic traits, a strategy that would exclude some earlier forms, particularly material attributed to *H. habilis* and *H. rudolfensis*. Tattersall (2017) further argues for the recognition of additional genera derived from phylogenetic analyses, in order to generate groupings that provide more informative comparative frameworks. Each of these proposals has advantages and are equally defensible. However, based on the arguments presented above, particularly comparisons with extant primates, some of the same issues can be addressed by expanding, rather than contracting, *Homo*. This suggestion does also come with several key disadvantages however (see Section 5), so the debate is likely to continue. What is clear from this present article, and those of Collard and Wood (2015), Tattersall (2017), and Villmoare (2018), is that taxonomic revision in homininan classification is needed.

Some authors have also approached these issues pragmatically by subdividing existing *Australopithecus* taxa in an effort to make monophyletic genera (Strait et al. 1997; Grine et al. 2006), or, in the case of *Kenyanthropus platyops*, by erecting a genus for new material that does not fall within any established homininan clade (Leakey et al. 2001). Indeed, when specimens cannot be accommodated within *Homo*, *Australopithecus*, or *Paranthropus* (but clearly fall within this larger homininan clade somewhere), the remaining options are either to erect additional genera or to retain a “wastebasket” grade taxonomy. Such splitting strategies therefore represent pragmatic attempts to preserve/create clades. However, they also risk producing substantial taxonomic instability, as genera may proliferate and change with each new analysis or fossil discovery if they are defined too narrowly. For the field as a whole, it is important either to fully adopt these additional genera (e.g., *Kenyanthropus* and *Praeanthropus*), and likely others, or to reject them all consistently (i.e., maintain a grade‐based australopith group). Recognizing some of these additional genera, but not others, while retaining a non‐monophyletic *Australopithecus*, may be more harmful than helpful, since it creates the appearance of phylogenetic structure that is not actually supported by phylogenetic analysis.

Defining a specific ecological or behavioral niche for the proposed expanded *Homo* is unnecessary, because it is justified as a well‐supported clade and as a substantial radiation that falls under a temporal depth comparable with other anthropoid primates. Although clearly it would be better, and more likely to be accepted by the community as a whole, if there were some key features that define this genus. There are some potential features that could be useful for descriptive purposes. These include habitual bipedalism, characterized by a uniquely derived pelvic‐lower limb complex for terrestrial locomotion, key dental traits (e.g., reduced canine dimorphism and thick‐enameled postcanine dentition), high cranio‐dental and postcranial variability reflecting dietary and ecological diversification. But these features are invariably found, to some degree, in the proposed earlier homininan genera, as well as in some other Miocene apes, so this is far from straightforward to provide a justification based on specific features (see limitations section below).

## 3 The Human Clade (Taxa More Closely Related to *Homo* Than to *Pan*)

Our broader clade, which includes all taxa more closely related to humans than to our closest living relatives (common chimpanzees and bonobos; *Pan*), is today commonly referred to as the tribe Hominini (hominins). This terminology, however, reflects a relatively recent shift (see summary in Almécija et al. 2021). Simpson (1945) divided Hominoidea into two families, Pongidae (all non‐human apes) and Hominidae (humans), and later revised this scheme (Simpson 1963), grouping all gibbons into *Hylobates* and all African apes into *Pan*. This arrangement positioned African apes as each other's closest relatives, then grouped all great apes together, followed by gibbons. As Goodman et al. (1990, 264) noted: “If only humans could be ignored, Simpson's (1963) classification was strictly cladistic”.

Although Simpson acknowledged evidence placing humans within the African great apes, he retained a separate family for humans, arguing that their behavioral and morphological divergence represented a distinct adaptive grade. In his view, African apes had remained relatively similar to one another, so even if humans were embedded within them, their uniqueness and speed of evolutionary change justified a separate family. This reasoning emphasized evolutionary grades, levels of complexity or adaptation, rather than strict phylogenetic relationships. This perspective remained dominant for several decades, but over time, particularly as cladistics became commonplace in evolutionary biology, researchers began to question this arrangement. The shift away from a family‐level designation for our section of the ape tree began in the 1980s, influentially with Groves (1986), who divided living apes into two families (Hylobatidae and Hominidae) and further split Hominidae into two subfamilies, Ponginae (orangutans) and Homininae (African great apes, including humans). Although several other, typically slight variations of this, taxonomies were also put forward (Wood and Richmond 2000; Andrews and Harrison 2005; and see Williams et al. 2023).

By the late 1990s and 2000s, most researchers had started to adopt a reduction from the family level (i.e., for all taxa closer to *Homo* than to *Pan*), with the tribal designation of hominin becoming dominant in the field. However, a minority continued to advocate a family level classification. Indeed, this practice has persisted, with some authors continuing to apply Hominidae where most would use Hominini all the way up to the present day (e.g., White et al. 2009; Tuttle 2014; Schwartz 2015; Tattersall 2017; Monson et al. 2026). Some researchers have argued that retaining this higher‐level taxonomy is necessary for various reasons. Schwartz (2015) contends that humans and their close relatives require family level status to permit finer subclade distinctions. Another justification for maintaining a family level human lineage is the diversity of fossil taxa it encompasses, even if only a single species survives today (Tattersall 2017). As Urciuoli and Alba (2023) note, this approach creates cladistic problems. Avoiding paraphyly would require assigning family level status to the *Pan* lineage and increasingly higher ranks to deeper nodes, quickly exhausting available categories, or leaving key, and useful, divergences unranked. Applying Hominidae exclusively to our seven‐million‐year‐old clade is therefore impractical in a strictly clade‐based taxonomy. Many authors who continue to use Hominidae for our lineage sidestep this issue by not specifying how their preferred taxonomic system accommodates the broader ape phylogeny. Presumably most continue with the previously used set up of a grade based combined non‐human great ape grouping. Some researchers have proposed slightly different solutions for this arrangement but still create non‐monophyletic groups (e.g., specifically for chimpanzees and gorillas; Pickford et al. 2022).

The current commonly used setup employed by most researchers today is largely adequate from a clade‐based perspective (unlike the genera discussed above). However, as Almécija et al. (2021) note, it still violates an important cladistic principle. This is partly due to a variation in how higher taxonomy is used for the apes, in that although now one family is typically stated for all great apes (Hominidae), the taxonomy used within the African great apes has varied after the change to a more cladistic friendly taxonomy, with the subfamily Homininae sometimes given to all African great apes, and other times just to the *Pan*‐*Homo* clade (and a separate subfamily for gorillas; Wood and Richmond 2000; Guy et al. 2003). When the three living African great ape genera are grouped together in the subfamily Homininae, often each is given its own tribe level status (Groves 2018). There are also slight variations in these setups (see Delson 2007). All these arrangements are perfectly reasonable and usable, but there is a crucial node in the tree missing, in that there is no taxonomic grouping for the living African great apes or because there is no grouping for the *Pan*‐*Homo* clade. Most commonly today, it is the node at the *Pan*‐*Homo* clade that is left unranked (see fig. 1 of Williams et al. 2023 which displays what is likely the most commonly used taxonomy for great apes today). This has sometimes led to confusion in the literature, in which the *Pan*‐*Homo* clade has been referred to as the tribe Hominini, at the same time as this tribe is also somehow also used to refer explicitly, and exclusively, to the *Homo* side of this tree.

Much of this taxonomic variation reflects the period during which the rank of family was being replaced by tribe for our side of the *Homo*‐*Pan* clade. Although there is now overwhelming evidence that *Homo* and *Pan* are sister taxa among living apes, this relationship was not yet firmly established during the 1980s to early 2000s. As a result, many of the emerging cladistic classifications avoided assigning a formal rank to the *Pan*‐*Homo* clade. Consequently, despite representing one of the most studied nodes in primate evolution today, the *Pan*‐*Homo* clade is still commonly left unranked, a legacy of this transitional period in primate taxonomy. It may also have been unpalatable at the time to reduce the human lineage from family directly to subtribe in one go. Lastly, there was still some ongoing debate about if we should allow some wiggle room on always using clades in our own taxonomy (for key discussions of these points toward the end of this crucial period see Andrews and Harrison 2005; Wood 2005; Delson 2007). Now, however, the relationships among the African great apes are fully resolved, and clade‐based taxonomy more universally insisted upon, making a solution straightforward. Hominini can be used for the entire *Pan*‐*Homo* clade, and Hominina for the human lineage specifically (all species closer to *Homo* than to *Pan*), and Panina for the chimpanzee clade. This keeps the higher‐level taxonomies the same and just changes our small branch of the tree to a lower taxonomic level. This allows each major branching event in ape evolution (from the perspective of living taxa) to have a major category in higher taxonomy. All apes (superfamily): Hominoidea; great apes (family): Hominidae; African great apes (subfamily): Homininae; *Pan*‐*Homo* clade (tribe): Hominini; human clade (subtribe): Hominina.

This means taxa commonly called hominins should be called homininans. Indeed, as noted by Strait (2013), although cladistically homininans is more accurate, hominin is typically used instead. Although not widely adopted, the more cladistically accurate/useful terminology has been used by some researchers (e.g., Ax 1985; Andrews and Harrison 2005; Diogo et al. 2012; Strait 2013; Carlson et al. 2021; Sarringhaus et al. 2022; Dolgova and Lao 2024). Given the roughly seven‐million‐year timeframe, the subtribe level is arguably also more appropriate for distinguishing these lineages relative to other anthropoid primate taxa. Most importantly however, this allows a cladistic crown‐stem framework whilst also keeping the key higher level taxonomic splits in the ape family tree (Ax 1985; Strait 2013), making it easier to discuss evolutionary history around the crucial earlier periods of our clades origins. For example, *Sahelanthropus*, *Orrorin*, and *Ardipithecus* can be discussed as potentially belonging to Panina, Hominina, or simply considered as crown or stem‐Hominini, or Homininae, more broadly.

## 4 Discussion

The taxonomic framework proposed here shares important similarities with the position advanced by Mayr, particularly in advocating a more inclusive genus *Homo*. However, several key elements of Mayr's argument have been undermined by subsequent fossil discoveries, most notably his contention that only a single homininan species lived at any given time. One aspect of Mayr's influential 1950 paper that remains particularly relevant is the exchange presented in its discussion section. Washburn, while agreeing that the proliferation of homininan genera should be avoided, questioned whether the human lineage might nevertheless be divided into two genera, a small‐brained “man‐ape” genus (*Australopithecus*) and a large‐brained, tool‐using genus (*Homo*). Mayr rejected this proposal, arguing that the fossil evidence available at the time indicated a single, continuously evolving lineage and therefore provided no basis for such a division. He further maintained that even if discrete morphological differences could be identified, they would not ordinarily justify generic separation in most animal groups. Human evolution is no longer interpreted as a single, steadily evolving lineage. The broader suggestion articulated by both authors has largely withstood the test of time however, that taxonomic inflation should be avoided, particularly at the genus level within a relatively brief evolutionary interval. From this perspective, the recognition of seven or more genera within Hominina, as is now common in contemporary classifications, is difficult to reconcile with the criteria typically employed for generic delimitation elsewhere in biology.

The present expanded *Homo* proposal is also in some ways similar to that of Goodman (1999), who reconstructed the ape family tree using genetic data and explicit cladistic methods. His framework remains valid, and in fact adhered more strictly to Hennig's (1966) original principle that taxa at the same hierarchical rank should represent clades that are equivalent in evolutionary age. On this basis, Goodman placed *Pan* within *Homo*. Although there is nothing intrinsically problematic about this suggestion, I here suggested a more recent and restricted *Homo*, limited to a single extant taxon. A genus that includes both chimpanzees and humans is unlikely to be especially useful in practice, even if such a classification also follows strict cladistic logic. In this context, the proposal to subsume *Paranthropus* and *Australopithecus* within *Homo* represents a more moderate and pragmatic attempt to bring homininan taxonomy into closer alignment with other fields. A genus‐level grouping extending back approximately 4–5 Ma still matches the temporal duration of genera observed in other primates (even if still on the recent side) and better accommodates growing evidence for extensive hybridization in anthropoid primates.

Finally, the taxonomic framework proposed here also bears some similarity to that of Robinson (1965, 1966). On the basis of the material then available, Robinson argued that there were minimal differences between the newly discovered specimens of *H. habilis* and the South African *Australopithecus* fossils, and therefore that separate genera were unwarranted. He accordingly proposed collapsing *Australopithecus* into *Homo*, though he notably retained *Paranthropus* as a separate genus. Robinson also drew attention to the problem of recognizing multiple genera within such a restricted span of geological time. However, his retention of *Paranthropus* as a distinct genus is difficult to reconcile with contemporary phylogenetic approaches, in which *Paranthropus* is almost invariably nested within the broader *Australopithecus*‐*Homo* clade (e.g., Dembo et al. 2015; Mongle et al. 2019; Martin et al. 2021; Irish and Grabowski 2021), that is, within the expanded *Homo* advocated here. However, direct comparison with these earlier debates is difficult today, given the substantial expansion of the homininan fossil record since Robinson's and Mayr's proposals.

The uniqueness and utility of homininan genera, especially *Homo* and *Australopithecus*, have been progressively eroded by new discoveries over recent decades. With each new find, different subsets of features have been treated as important genera criteria, only to be revised again with subsequent material. Since the mid‐twentieth century, an expanded fossil record has shown a far more complex evolutionary pattern than once suspected, characterized by mosaic trait distributions across lineages. Moreover, it has become increasingly clear that “wastebasket” taxa defined by one or a few traits, such as brain size, locomotion, or tool use, do not map neatly, or even plausibly, onto phylogenetic reconstructions of homininans.

This process has led to continual reclassification of previously known fossils (see overview in de Ruiter et al. 2017), to the point that the distinctions between these genera have become trivial at best, and phylogenetically misleading at worst. This problem extends beyond morphology. The supposed adaptive zone of *Homo* (as commonly defined today) and the characters used to infer it (see Wood and Collard 1999) appear to have emerged rather piecemeal (Antón et al. 2014; Holliday 2012; Kimbel and Villmoare 2016), undermining the coherence of the *Homo* adaptive zone as it is usually construed (de Ruiter et al. 2017). For example, Strait (2013) identified four traits as behaviorally important adaptations of *Homo*: (1) an enlarged brain; (2) the manufacture and use of stone tools; (3) fully committed terrestrial bipedality; and (4) reduced reliance on processing highly resistant foods in the oral cavity. Subsequent fossil discoveries, most notably *H. floresiensis* and *H. naledi*, pose a serious challenge to this model. *Homo naledi* fails to conform to these criteria in all respects except potentially tool use, and even this trait is now widely recognized in other homininan genera (and indeed a growing list of extant primates). In particular, *H. naledi* possessed a small brain (Berger et al. 2015; Hawks et al. 2017) and showed key adaptations for climbing (Kivell et al. 2015; Feuerriegel et al. 2017; Chapman et al. 2025). Although its dentition is relatively small, it shares features with more robust australopiths, including robust aspects of individual teeth (Kupczik et al. 2019; O'Hara 2021; Mahoney et al. 2024). *Homo floresiensis* poses similar challenges to the supposed key *Homo* characteristics (e.g., Pietrobelli et al. 2025). Similar issues are well known and discussed with regards to early *Homo* taxa (see Kimbel and Villmoare 2016; Zanolli et al. 2026).

The growing realization of the mosaic nature of homininan evolution therefore provides one of the strongest arguments for now being the right time for subsuming *Australopithecus* and *Paranthropus* within *Homo*. Indeed, mosaic evolution is evident across many anatomical systems that have been studied, not only when comparing different regions of the body, but also within individual systems such as the dentition and the foot (e.g., DeSilva et al. 2019; Grine et al. 2026; Orr et al. 2026). These characteristics, presumably through processes such as rapid multiple recent branching of lineages, homoplasy, convergence and potentially hybridization, is also found in other anthropoid primates that show a similar large radiation on similar time scales. The Papionini examples discussed above are a perfect example of this.

It has been suggested that, in exceptional cases, it may be necessary or useful to combine cladistic and patristic information (Harrison 1993). It has also been noted that the strict requirement of monophyly in cladistic classifications can result in two closely related and morphologically very similar fossil taxa being placed in very different taxonomic categories (Simpson 1975; Harrison 1993). Although strict cladistics maintain that grade‐based concepts are never appropriate, even proponents of more flexible approaches have emphasized important constraints. Harrison (1993), for example, argued that such departures should not be applied to groups for which there is clear evidence of paraphyly or polyphyly, and that any non‐monophyletic grouping used in the fossil record must be explicitly identified and justified. Neither of these conditions is met in the homininan fossil record. The current arrangement of genera, and of higher taxonomic levels (for those continuing to use hominid for our lineage), therefore violates the criteria even for those who are willing, in limited circumstances, to relax strict cladistic rules. As a result, biological anthropology occupies an unusual position in that although most active researchers would, in principle, favor revising the existing taxonomic framework, there appears to be little practical momentum toward implementing such change, likely because there is no obvious solution.

Indeed, in practice, non‐monophyletic groupings continue to be regularly used, often with the awareness that they are not clades (although not always). Moreover, many studies explicitly claim to adopt cladistic approaches while simultaneously displaying and using non‐cladistic groupings, without acknowledging or justifying this discrepancy. We cannot have it both ways as a field; at a minimum, it is necessary to state explicitly when and why grade‐based (or other non‐monophyletic) groupings are being used. The framework proposed here allows for the recognition of what is commonly proposed to be a well‐supported clade. *Australopithecus anamensis* is almost always regarded as the sister taxon to all other *Australopithecus* plus *Paranthopus* and *Homo (*and *Kenyanthropus*) (Hammond and Ward 2013; Strait 2013; Dembo et al. 2015; Mongle et al. 2019; Martin et al. 2021). This human‐*A. anamensis* clade and its relative consensus is rare in paleoanthropology, and it provides an unusual opportunity to establish a taxonomic grouping with a defensible cladistic basis. This therefore represents a reasonable attempt to define a clade and to place this genus within a temporal framework that is more comparable to those used for other catarrhine primates.

Earlier putative homininans, including *Sahelanthropus*, *Orrorin*, and *Ardipithecus*, remain variably contentious in their placement within or outside Hominina (e.g., Andrews and Harrison 2005; Strait 2013; Macchiarelli et al. 2020; Wood et al. 2020; Williams et al. 2026). While some of these taxa have been proposed as potential close relatives of the common ancestor of the expanded *Homo*, there is little evidence that any fall within this clade (but see Haile‐Selassie et al. 2025). Importantly, this framework does not exclude these genera from Hominina more broadly, as the expanded *Homo* need not encompass the entirety of Hominina evolution.

## 5 Limitations, Drawbacks, and Further Considerations

The principal limitation of the proposed expansion of the genus *Homo* is the loss of taxonomic information. Taxonomy serves not only to provide labels for biological groups but also to communicate information about their evolutionary history, morphology, and ecology. By subsuming *Australopithecus*, *Paranthropus*, and *Homo* within a single genus, some of this information is inevitably obscured. In particular, most researchers would likely agree that, despite their imperfections, the genera *Homo* and *Paranthropus* currently convey meaningful information about two sympatric lineages that followed markedly different evolutionary paths and exhibited important anatomical and behavioral differences. Consequently, many will view the taxonomy proposed here as chucking the baby (*Homo* and *Paranthropus*) out with the bath water (*Australopithecus*). This criticism has considerable merit and will make this proposal unlikely to gain momentum. Although the proposals by Wood, Collard, and Tattersall introduce a greater number of genera (or else increase the size of grade‐genera), they have the advantage of explicitly recognizing what were clearly major transitions within human evolution, including when large‐brained and fully committed terrestrial *Homo* evolved.

One possible response to this critique is that additional taxonomic ranks, such as subgenera or species groups, could be used to retain information within a broader *Homo* framework. For example, a subgenus could be applied to the recent large‐brained members of *Homo*, which are regularly recovered as a clade and has clear shared behavior/anatomical features, while taxa currently assigned to *Paranthropus* could similarly be accommodated as a subgenus (e.g., *Homo* (*Paranthropus*)). A comparable structure could be implemented using species groups, as is common in extant primate taxonomy (e.g., *Macaca*, *Trachypithecus*), thereby preserving meaningful biological and evolutionary distinctions. However, such approaches remain less satisfactory than the use of distinct genera that more clearly reflect these major evolutionary, morphological, and ecological differences.

From a broader primate perspective, the recognition of seven or more genera within Hominina is difficult to reconcile with conventional standards of generic delimitation. It has previously been argued that, given the relatively short temporal span of the homininan fossil record, such proliferation is difficult to justify, and that smaller brained hominins may instead be more appropriately accommodated within a more inclusive genus, effectively a grade‐based arrangement (e.g., *Australopithecus*; Foley 2013). I agree with Foley regarding the underlying problem, although I differ in the proposed solution of maintaining a grade‐based system for some (but not all) taxa. In my view, this approach risks obscuring evolutionary relationships and processes, and may inadvertently do more harm than good in the literature. At the same time, achieving a framework that satisfies the competing demands of clades, taxonomic stability, and meaningful biological information remains challenging. I think most researchers would agree that ideally, all genera would represent well supported clades, either *Homo* as currently defined or reduced to only later large‐brained *Homo*, and *Paranthropus* would be retained as a distinct genus, and the total number of genera would remain limited as much as possible. At present, however, these different criteria appear difficult to reconcile, suggesting that some form of compromise is unavoidable. Here I propose a solution that addresses some, but not adequately all, of these issues.

Another potential objection to incorporating *Paranthropu*s and *Australopithecus* into *Homo* is that this move simply displaces issues backward in time. Under such a scheme, hybridization, mosaic evolution, and overlapping traits might instead be expected to be enhanced between an expanded *Homo* and other homininan (or hominin more broadly) taxa. The advantage of the proposed framework, however, is that it aligns taxonomic boundaries more closely with the temporal cut offs used in other primate lineages. Genera originating deeper in time likely experienced interbreeding with closely related lineages during their initial diversification, but these effects should start to diminish after the first couple of million years as reproductive isolation increased. By contrast, a genus originating around ~2–3 Ma would have had little or no period in which hybridization was rare, given patterns observed among extant catarrhine primates (see above). Consequently, very young genera are especially problematic, particularly in the fossil record where direct genetic evidence may be unavailable for untangling such complexities. Collapsing such taxa into an expanded *Homo* therefore reduces the risk of later taxonomic change.

Attempts to enforce monophyly through splitting taxa (the opposite of the current proposal) would generate an increasing number of genera and likely frequent taxonomic reassignment, rather than a stable and coherent classification. Perhaps the furthest extreme of such splitting proposed in recent years, Tattersall (2026) has suggested that not only the broader homininan tree, but also taxa currently placed within *Homo*, would benefit from further generic subdivision. However, such an approach is difficult to reconcile with genetic evidence from other primates and with the instability observed across competing phylogenetic analyses (i.e., stable clades are hard to identify). If a strategy of increasing the number of homininan genera is adopted, it should be applied consistently so that all genera represent clades rather than a mixture of clade‐ and grade‐based groupings. A taxonomy combining clade‐based genera with grade‐based ones can be misleading, as it risks implying phylogenetic relationships that are not actually supported. A good example is *Kenyanthropus platyops*. This genus has gained broad usage within paleoanthropology. The issue is not its creation, which could be appropriate under clade‐based principles, but rather its coexistence with continued uncritical use of a grade‐based *Australopithecus*. Consequently, increasing the number of genera does not necessarily improve phylogenetic resolution, or indeed add any more information, under the current homininan taxonomic framework. The only clear alternatives are either to subdivide *Australopithecus* (and potentially *Homo*) into an even larger number of smaller genera (Tattersall 2017, 2026), or, as proposed here, to recognize a single large Hominina radiation over the past 4–5 Ma.

The traits used to diagnose an expanded *Homo* are not always straightforward, even though the clade as a whole is defined by a broader suite of shared synapomorphies. For example, several fossil apes outside Hominina exhibit substantial canine reduction and a non‐sectorial lower third premolar, converging to varying degrees on homininan dental morphology (e.g., *Indopithecus*, *Gigantopithecus*, *Ouranopithecus*, *Lufengpithecus*; Simons and Chopra 1969; Koufos and de Bonis 2006; Xu and Lu 2007; Wang 2009; Zhang and Harrison 2017; see discussion in Zanolli et al. 2019). Miocene apes also display a wide range of locomotor diversity, and there is potential for partial bipedal adaptations to have evolved independently. Moreover, given the broad temporal range estimates of when the last common ancestor of chimpanzees and humans lived, various fossil taxa may exhibit subsets of traits associated with the homininan phenotype. Thus, in addition to early putative homininans such as *Sahelanthropus*, *Orrorin*, and *Ardipithecus*, other fossil apes may display one or more key features traditionally linked to this clade. Consequently, although there is little evidence that any of these taxa belong to the expanded *Homo* proposed here, it may not be straightforward to identify an umbrella ecological, behavioral, or particular morphological criterion that uniquely unites the genus in the sense often done for justifying genera. However, such an adaptive niche is not strictly necessary for defining this genus as it fits both the timescale based criteria and a well‐supported clade.

## 6 Conclusion

The central argument of this paper is not that the specific taxonomic solution proposed here is necessarily the best one, but rather that the current treatment of homininan genera is increasingly difficult to justify. As a field, paleoanthropology continues to employ a large, grade‐based *Australopithecus* while simultaneously favoring clade‐based genera (and other higher‐taxonomic levels) both within and outside this group. New genera have at times been proposed based on relatively limited ecological or behavioral distinctions, while growing fossil evidence is increasingly blurring some of the boundaries between already well‐established genera such as *Homo* and *Paranthropus*. The result is a taxonomic framework that is not just difficult to use, but at times actively misleading.

The proposal advanced here represents one possible solution to some of these problems. Given its limitations and the inevitable loss of some taxonomic information, it will likely not prove widely acceptable. Nevertheless, the issues discussed are not new, and recent discoveries have only amplified them. Therefore, there is an increasing need for greater consistency in how genera are defined and applied across the homininan fossil record. Ultimately, higher‐level taxonomy will always contain an element of arbitrariness, and debate over the appropriate classification of homininans is unlikely to disappear. However, the discipline should, at minimum, strive for classifications that are phylogenetically coherent and consistent with the principles of modern evolutionary biology. If non‐monophyletic groups are retained, their use should be explicit and clearly justified whenever used. It is also important to strive for genera that are applied consistently and justifiably across the broader homininan tree. The growing fossil and genetic record provides an opportunity not only to reassess long‐standing phylogenetic assumptions but also to develop a more coherent and defensible framework for understanding human evolution.

## Author Contributions

**Ian Towle:** conceptualization, writing – original draft, writing – review and editing, investigation, project administration, resources.

## Conflicts of Interest

The author declares no conflicts of interest.

## Data Availability

Data sharing not applicable to this article as no datasets were generated or analysed during the current study.
